# Amplification cycles through innate lymphoid cells at the onset of lupus nephritis

**DOI:** 10.3389/fimmu.2026.1756560

**Published:** 2026-03-12

**Authors:** Rosa L. Kreider, Stylianos-Iason Biniaris-Georgallis, Bastian Grothey, Antigoni Triantafyllopoulou, Lisa M. Steinheuer, Kevin Thurley

**Affiliations:** 1 Biomathematics Division, Institute of Experimental Oncology, University Hospital Bonn, Bonn, Germany; 2 Department of Nephrology and Medical Intensive Care, Charite-Universitätsmedizin Berlin, Berlin, Germany; 3 Berlin Institute of Health at Charite-Universitätsmedizin Berlin, Berlin, Germany; 4 Macrophages in Chronic Inflammation, German Rheumatism Research Centre (DRFZ), Berlin, Germany; 5 Bonn Center for Mathematical Life Sciences, University of Bonn, Bonn, Germany

**Keywords:** autoantibodies, autoimmune disease, cell-cell communication, mathematical model, single-cell transcriptomics, response-time modeling, NZB/W F1 mice

## Abstract

Disease progression in autoimmune conditions such as systemic lupus erythematosus (SLE) is highly heterogeneous, and the cellular and molecular mechanisms driving disease-onset dynamics remain incompletely understood. Here, based on single-cell transcriptomics data on lupus-prone NZB/W F1 mice, we derived a mathematical cell-cell interaction model recapitulating early dynamics of innate immune cells in lupus nephritis. We identified a diverse landscape of tissue-associated ILC and vessel-associated NK cell populations. We conceived a scalable mathematical framework for analysis of immune-cell interaction dynamics. A specific model formulation considers ILC as amplifiers of inflammatory processes in the presence of autoantibodies in lupus-prone individuals. Systematic model analyses highlight the impact of positive feedback loops and spontaneous inflammatory events or environmental stimuli, and the timing-dependent effectiveness of depletion therapies. Additionally, our model links the critical role of ILC populations to hallmarks of SLE such as highly heterogeneous disease dynamics. Overall, our findings lay the groundwork towards a mathematical model of immune-tissue cellular crosstalk, enabling quantification of disease severity and prediction of responses to biologic treatments in autoimmune diseases.

## 1 Introduction

Interactive dynamics of diverse immune-cell populations shape the type and strength of immune responses by means of a collective decision-making process. While late or ineffective immune responses are causal for susceptibility to infections and many cancer entities, strong immune responses impose the risk of developing autoimmune conditions and immunopathology. Despite the large body of knowledge on cellular phenotypes and their dynamic interplay in both the adaptive and the innate branch of the immune system, a quantitative and mechanistic understanding of immune-cell networks is only beginning to emerge (1–6).

Systemic lupus erythematosus (SLE) is a chronic inflammatory autoimmune disease primarily affecting young women (7, 8). The disease can involve multiple organs. Its renal manifestation, known as lupus nephritis, can be life-threatening and is associated with significant morbidity and mortality. Pathophysiological mechanisms include impaired clearance of immune complexes and apoptotic material (7, 9, 10). Up to 80% of SLE patients exhibit elevated type I interferon (IFN-I) activity, which correlates with more active disease (11–13), and large-scale transcriptomics studies revealed IFN-response gene signatures in all major compartments of myeloid cells and lymphocytes (13–15). Lupus nephritis is characterized by autoreactive B cell-derived antinuclear antibodies, followed by immune-complex deposits, subsequently leading to inflammation and tissue damage. Genome-wide association studies have identified more than 50 risk loci for SLE susceptibility (16, 17), many of which are related to the major histocompatibility complex and thus may explain the presence of autoantibodies in SLE patients. However, data suggest that disease onset is highly heterogeneous and associated with additional factors such as infectious agents and environmental factors.

Recently, innate lymphoid cells (ILC) (18) were proposed as another important contributor to SLE pathophysiology (19, 20). ILC are a heterogeneous group of innate immune cells derived from common lymphoid progenitors, with immunomodulatory and cytotoxic functions that vary by subtype (18). NKp46^+^ ILC include conventional NK cells as well as ILC1 and NKp46^+^ ILC3, in the following termed helper ILC. To functionally assess their role in disease onset, we employed NZB/W F1 mice (21), a murine lupus nephritis model that closely mirrors several hallmarks of human lupus nephritis. Surprisingly, despite the association of SLE to auto-antibodies and the adaptive immune system, we demonstrated (20) that NK cells and helper ILC are pivotal in the autoimmune mechanisms driving lupus nephritis, by promoting the expansion of disease-associated macrophages and exacerbating epithelial cell injury.

Here, starting from quantitative analysis of a rich single-cell transcriptomics data set derived from NZB/W F1 mice under poly(I:C) treatment, we developed a specific mathematical model of the course-of-events leading to onset and chronification of lupus nephritis. For that purpose, we derived a flexible and scalable mathematical modelling framework for conceptual analysis of cell-state dynamics and cell-cell interactions in the immune response. We focused on NK cells and helper ILC and their interactions with myeloid cells and tissue cells in the kidney, and on how these processes are modulated by the presence of autoantibodies in lupus-prone individuals. Our model simulations reconcile observations such as the strong role of the ILC compartment as amplification module at disease-onset and the highly heterogeneous dynamics of autoimmune diseases with the established concept of SLE as an interferon and autoantibody-mediated condition.

## 2 Results

### 2.1 Single-cell transcriptomics reveal a diverse landscape of activated ILC populations in nephritic mice

**Figure 1:**
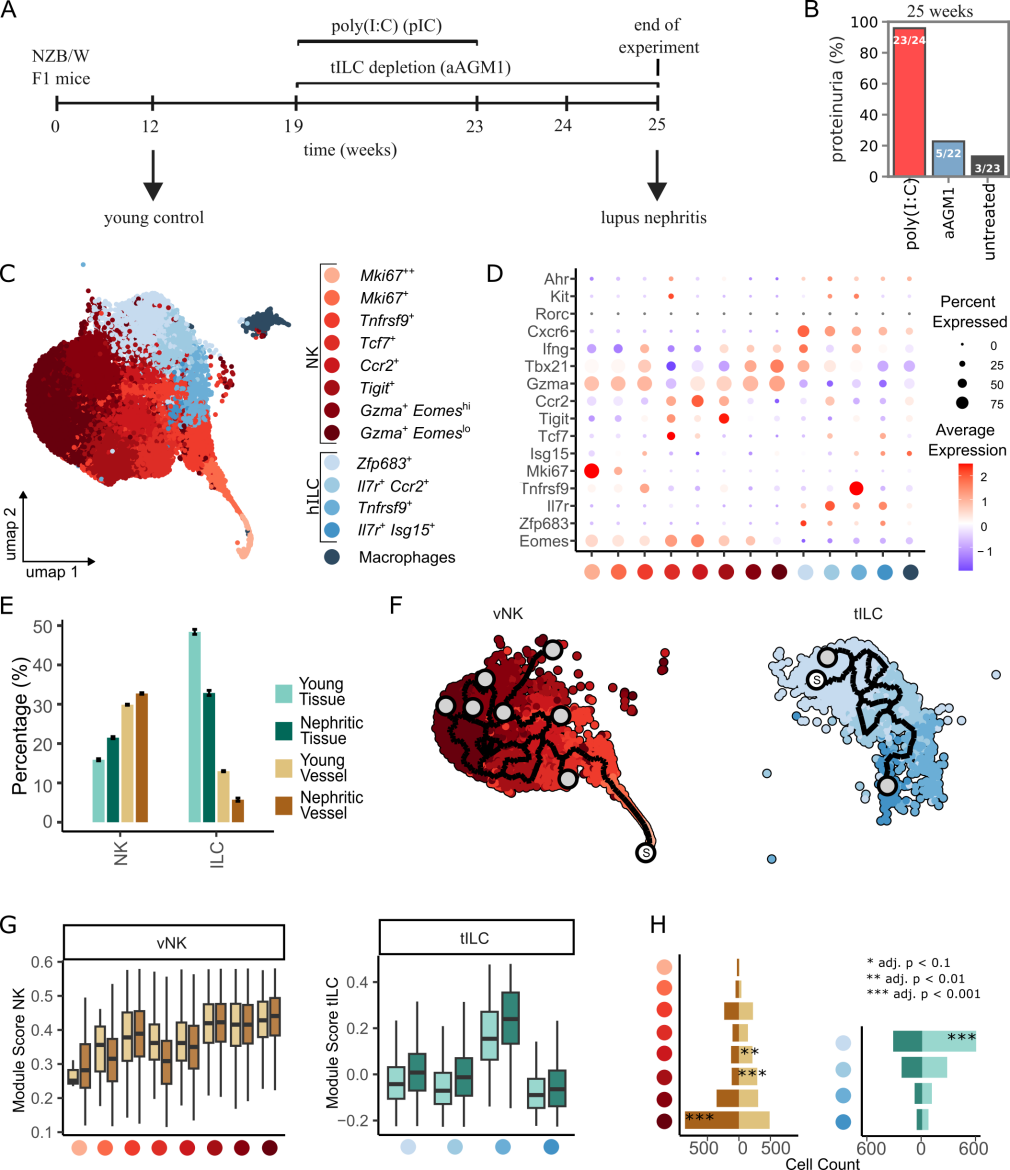
Single-cell transcriptomics analysis of ILC populations in the kidney of lupus-prone NZB/W F1 mice. **(A)** Experimental outline and assessment of proteinuria. NZB/W mice were treated with poly(I:C) in weeks 19 to 23, and samples were collected at week 12 (control) and at week 25 (disease). For assessment of proteinuria, also mice under ILC depletion using aAGM1 and mice without poly(I:C) treatment were assessed. **(B)** Percentage of mice that developed proteinuria after 25 weeks. Numbers on the bars indicate the ratio of mice with positive proteinuria measurements to the total number of mice. **(C, D)** UMAP visualization and cell-type annotation of NKp46+ ILC populations based on selected marker genes, a full list of data-derived signature genes is provided in **Supplementary Table 1**. **(E)** Cell-type abundance after down-sampling to the smallest sample analyzed. Error bars indicate the standard deviation of 1000 independent down-sampling runs. **(F)** Trajectory analysis after down-sampling to the smallest sample analyzed. The starting point is manually selected and indicated by the letter “S”. **(G)** Activation status based on selected gene-sets. Each box-plot represents the module-score of that gene-set, color-code as in panels **(C, E)**. **(H)** Absolute cell-counts from down-sampled datasets. P-values are computed using permutation tests, color-code as in panels **(C, E)**.

To assess ILC populations at the onset of lupus nephritis, we analyzed data from lupus-prone NZB/W F1 mice obtained upon immune-stimulation by injection of poly(I:C) (**Figure 1A**). Almost all analyzed mice showed onset of proteinuria, which was reduced to 23% under aAGM1 treatment causing depletion of NKp46^+^ cells primarily in tissue (20), and 13% in the absence of poly(I:C) stimulation within this timeframe (**Figure 1B**). Of note, those experiments were routinely terminated at 25 weeks of age, before the onset of end-stage renal disease (ESRD). Earlier studies showed that in the absence of poly(I:C), female NZB/W F1 mice have a very heterogeneous lifespan in the range of 21–57 weeks (36.9 weeks mean, ~ 7.2 weeks s.d.) and are prone to onset of proteinuria at 20–44 weeks of age (22–24). Overall, the effect of aAGM1 treatment on the onset of proteinuria in poly(I:C)-treated NZB/W F1 mice substantiated a strong role of NKp46^+^ ILC at the onset of disease. Hence, we investigated the transcriptional landscape of NK cell and helper ILC populations of young (before poly(I:C) treatment) and nephritic mice (after poly(I:C) treatment), based on data on tissue cells from Ref (20), supplemented by acquisition of additional data on vessel-associated cells, derived by injecting anti-CD45 labeling antibodies intravenously before analysis.

By clustering of the single-cell data and applying a curated panel of ten lineage and activation markers, we annotated 13 cell-states and uncovered functional heterogeneity within both the NK cell and helper ILC compartments (**Figures 1C, D**; **Supplementary Table 1**; **Supplementary Figure 1A**). Within the NK cell populations, two clusters stood out for their high expression of *Mki67*, indicative of their proliferative capacity, while two large NK cell clusters exhibited robust *Gzma* expression, reflecting a cytotoxic effector phenotype. These NK cell subsets further differed in their expression levels of *Eomes*, suggesting the presence of distinct activation levels. Beyond conventional activation signatures, we identified a discrete NK cell cluster with elevated expression of the immune-checkpoint receptor *Tigit*, suggesting a subset potentially involved in immune regulation or suppression. To further assess lineage identity within the helper ILC compartment, we examined canonical ILC1- and ILC3-associated markers, including *Tbx21*, *Ifng*, and *Cxcr6* for ILC1, as well as *Rorc*, *Kit*, and *Ahr* for ILC3 identity. Although subsets showed enrichment of ILC1-associated genes, *Rorc* expression was limited and not consistently detectable across clusters, and we therefore analyzed the helper ILC population as a unified compartment. Though smaller in number of cells, the helper ILC population demonstrated marked heterogeneity: one cluster prominently expressed *Tnfrsf9*, a marker of cellular activation, while another large subset was defined by high *Zfp683* expression, a transcription factor associated with tissue residency and long-term retention within the local microenvironment. We further analyzed the expression of key cell–cell communication molecules and found that differential expression of *Ifng* and chemokines such as *Ccl5* highlighted diversity in the activation status at the onset of disease, as expected (**Supplementary Figure 1B**; **Supplementary Table 1**). Moreover, *Csf1* and *Csf2* were distinctly expressed in the *Tnfrsf9*^+^ ILC cluster, indicating a potential role in supporting the population expansion of myeloid cells.

Next, we analyzed the relative contribution of the identified NK cell and helper ILC subsets (**Figure 1E**; **Supplementary Figure 1C**). While NK cell populations outnumbered helper ILC in vessels, the latter clearly dominated in tissue, and hence we focused on helper ILC in tissue (tILC) and NK cells in vessels (vNK) for further analysis. To gain insight into the dynamic progression of activation states, we employed pseudotemporal ordering (**Figure 1F**), and we found that the *Gzma*^+^ subset was at the end of the NK cell activation trajectory. We then quantified the effector potential through gene-module scoring, using NKp46 activation signatures for tILC and degranulation signatures for vNK cells (**Figure 1G**; **Supplementary Table 1**). Effector potential progressively increased along the activation trajectory in vNK cells. Interestingly, in tILC, the third activation state based on trajectory analysis displayed the highest effector potential, and effector potential was relaxed back to the level of the tissue-resident Zfp683^+^ subpopulation in the final state of the trajectory. In vNK cells, the cell-state with highest activation level was enriched in nephritic compared to young samples (**Figure 1H**), while in tILC, the tissue-resident cell-state with low effector potential was enriched in healthy samples. To evaluate ILC activation patterns also in human disease, we reanalyzed a published single-cell RNA-seq dataset obtained from lupus nephritis patients and applied our activation-signature framework to NK cell and ILC populations. Clustering resolved three NK cell and two ILC subpopulations (**Supplementary Figures 2A, B**), each showing large cellular heterogeneity in activity scores and a tendency to elevated activity in specific subpopulations (**Supplementary Figures 2C, D**). Gene-level expression patterns of activation-associated markers further distinguished these subclusters (**Supplementary Figure 2E**), and the NK cell and ILC states showing highest activation signatures corresponded to their counterparts in the murine dataset (**Supplementary Figure 2F**). Hence, although the murine analysis provided higher resolution due to targeted enrichment and higher cell numbers, the overall activation patterns were consistent with patient-derived cells.

Taken together, the identification of a diverse landscape of ILC populations with various effector functions in lupus nephritis supports a pivotal role for activated tILC and vNK cells at the onset of autoimmune disease.

### 2.2 Mathematical model of cell-cell interactions driving chronic inflammation in lupus nephritis

**Figure 2:**
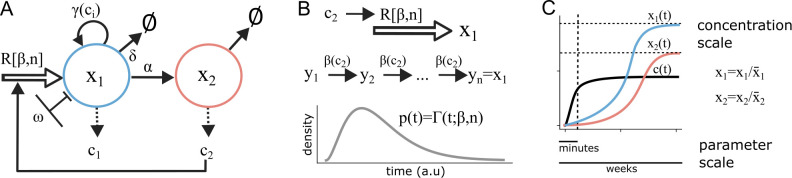
Mathematical description of interactive cell-population dynamics. (**A**) Minimal example showing all processes considered: cell infiltration described by an Erlang distribution R[β,n], cell-state changes (α), cell-death (δ), cell-proliferation (γ(c_i_)) up to a carrying capacity (ω), cytokine secretion and feedback. **(B)** Response-time modelling framework. The unfilled arrow represents a delayed input process described by Erlang-distributed stochastic variables, which can be replaced by a linear chain of reactions reaction rate β. **(C)** Visualization of non-dimensionalization and scaling to intrinsic units for groups of cell-types and cytokines.


For model development, we established a general framework that extends established network motifs of cell–cell interactions, such as macrophage–tissue cell communication and cytokine-mediated growth control (25–27), to larger network topologies. The guiding principles of our framework can be illustrated using a minimal example with two cellular and one cytokine species (**Figure 2A**). The framework incorporates cell-infiltration and cell-cell communication via cytokines as well as delayed production-terms representing cross-compartment processes such as immune-complex formation. The latter are implemented using our previously described response-time modelling framework based on the linear-chain trick (**Figure 2B**) (25, 28), to consider intermediate processes without explicitly describing all intermediate species in detail. In more general terms, we arrived at a coupled equation system for cell-states $x_{i}$ and cytokine species $c_{j}$ of the form

$$\begin{array}{l} \frac{dx_{i}}{dt}\text{ = }R[\beta_{i}\left(.\right),n_{i}]+\sum_{n=1}^{N_{cell}}\left(\alpha_{\text{in}}\left(.\right)(x_{n}-x_{i})\right)+\\ \left[\gamma_{i}\left(.\right)\left(1-\frac{x_{i}}{\omega_{i}}\right)-\delta_{i}\right]x_{i},\;i=1\ldots N_{cell} \end{array}$$

$$\frac{dc_{j}}{dt}\text{ = }\sum_{n=1}^{N_{cell}}q_{nj}x_{n}-\lambda_{nj}h\left[c_{j},K_{j}\right]x_{n}-\eta_{j}c_{j},\;j=1\ldots N_{cyt}.$$

Here, R[β,n] describes the delay represented in terms of our response-time modelling framework and $h\left[c,K\right]\left(t\right)=\frac{c{\left(t\right)}^{3}}{K^{3}+c{\left(t\right)}^{3}}$ is a Hill-type function. The coefficients $\beta_{i},\alpha_{ij},\gamma_{i}$ may depend on all cytokines and cell types, by relations of the form $\beta_{i}=\beta_{i,0}\sum_{n}h\left[c_{n},K_{\text{in}}\right]\sum_{m}h\left[x_{m},K_{\text{im}}\right]$. Finally, using non-dimensionalization, we formulate models in terms of intrinsic units for each group of cytokines and cell-types (**Figure 2C**), to reduce the parameter space and facilitate systematic analyses focusing on network topology (Supplementary Text). That modeling work-flow allowed mathematical formulation and systematic analysis of the involved cell-cell interaction systems that are at the core of immunological control circuits.

**Figure 3:**
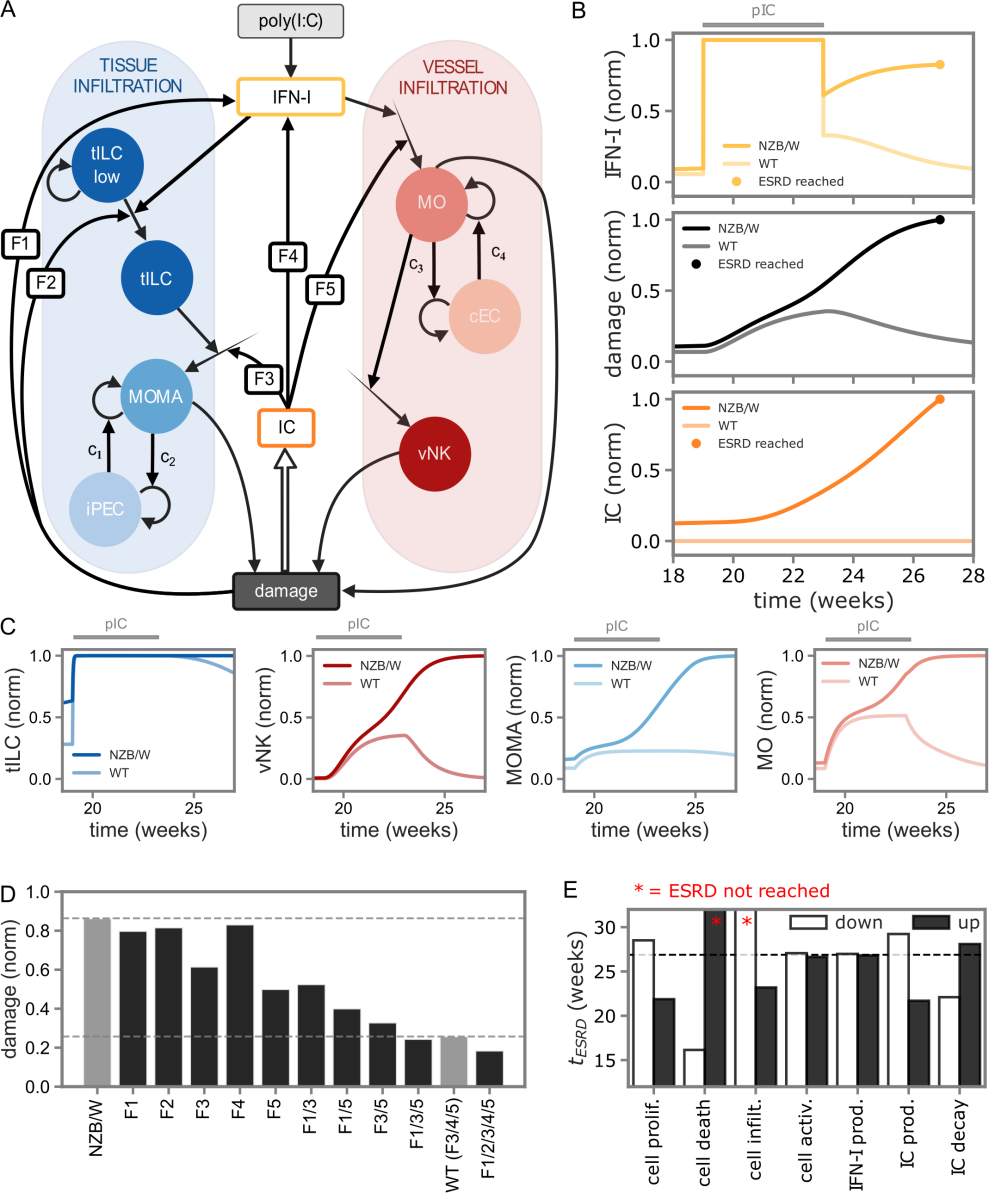
Mathematical model of innate immune-cell interactions driving chronic inflammation in lupus nephritis. **(A)** Schematic of the specific model, structured into tissue and vessel compartments. Delayed processes described by Erlang distributions (cf. **Figure 1B**) are represented by unfilled arrows. Labels F1-F5 denote the positive feedback loops considered in the model. **(B, C)** Simulated kinetics for NZB/W and WT conditions under poly(I:C) treatment. Simulations are stopped after reaching 95% of the maximal damage value, which is identified with ESRD. **(D)** Feedback-loop analysis. Simulations assess the impact of eliminating individual feedback loops on the damage value at 25 weeks. Feedback loops are labeled as in panel **(A)**. Eliminating F3/4/5 corresponds to the wild-type mice condition, in which immune-complex mediated feedback is absent. **(E)** Sensitivity analysis of model parameters by up- and down-regulation of default parameter values by 20%. The y-axis shows the time to onset of ESRD.

Next, we derived a specific mathematical description of innate immune-cell interactions (**Table 1**) (29–35) at the onset of lupus nephritis, composed of two model compartments: nephritic tissue and vessel-associated cells (**Figure 3A**). Our model design is focused on IFN-I and immune complex-mediated positive feedback loops linking immune-cell recruitment to progressive tissue damage (36–38), see Supplementary Text for details. Specifically, upon poly(I:C) stimulation inducing IFN-I release, tissue-residing tILC populations with low effector potential (tILC low) are transformed to more active populations (tILC), and stimulated monocytes (MO) arrive in the vessels. NK cells immigrate to the nephritic vessel compartment along with increasing MO numbers, and tILC populations drive an increase in monocyte-derived macrophages (MOMA) in tissue. In disease, a population of lupus-nephritis associated intermediate parietal epithelial cells (LN-iPEC) emerge, and they produce chemokines such as CCL2, CCL5 and CXCL16 and hence are suggested to play a role in recruitment of macrophages. Further, in disease, a population of lupus nephritis-associated capillary endothelial cells (LN-cEC) was detected. Therefore, we chose iPEC and cEC as representative regulators of the capacity for tILC and vNK-cell driven presence of MOMA and MO cells, which together with vNK cells contribute directly to tissue damage. To reflect disease-onset in lupus-prone individuals, we considered genetic predisposition to the presence of autoantibodies and damage-associated immune-complex deposition.

**Table 1:** Model equations.

Variable	Name	Equations
x_1_	tILC low	$\frac{dx_{1}}{dt}\text{= }\gamma \left(1-\frac{\left(x_{1}+x_{2}\right)}{\omega }\right)\left(x_{1}+x_{2}\right)-\alpha min\left\{h\left[c,K_{1}\right]+\zeta_{2}h\left[x_{8},K_{2}\right],\;1\right\}x_{1}-\delta x_{1}$
x_2_	tILC	$\frac{dx_{2}}{dt}\text{= }\alpha min\left\{h\left[c,K_{1}\right]+\zeta_{2}h\left[x_{8},K_{2}\right],\;1\right\}x_{1}-\delta x_{2}$
x_3_	MOMA	$\frac{dx_{3}}{dt}\text{= }\beta min\left\{h\left[x_{2},\;K_{2}\right]+\zeta_{3}h\left[x_{9},K_{2}\right],\;1\right\}+\gamma h\left[c_{2},K_{2}\right]x_{3}-\delta x_{3}$
x_4_	iPEC	$\frac{dx_{4}}{dt}=\left(\beta +\gamma h\left[c_{1},K_{2}\right]x_{4}\right)\left(1-\frac{x_{4}}{\omega }\right)-\delta x_{4}$
x_5_	vNK	$\frac{dx_{5}}{dt}\text{= }\beta h\left[x_{6},K_{3}\right]-\delta x_{5}$
x_6_	MO	$\frac{dx_{6}}{dt}\text{= }\beta min\left\{\zeta_{5}h\left[x_{9},\;K_{2}\right]+\vartheta h\left[c,\;K_{1}\right],\;1\right\}+\gamma h\left[c_{4},K_{2}\right]x_{6}-\delta x_{6}$
x_7_	cEC	$\frac{dx_{7}}{dt}=\left(\beta +\gamma h\left[c_{3},K_{2}\right]x_{7}\right)\left(1-\frac{x_{7}}{\omega }\right)-\delta x_{7}$
x_8_	damage	$\frac{dx_{8}}{dt}\text{= }\mu_{1}\left(x_{3}+x_{5}+x_{6}\right)-\nu_{1}x_{8}$
x_9_	IC	$\frac{dx_{9}}{dt}\text{= }R\left[\mu_{2},n\right]x_{8}-\nu_{2}x_{9}$
c	IFN-I	$c\text{= }min\left\{q_{0}+q_{1}1{\left(t\right)}_{19\leq t\leq 23}+q_{2}\left(\zeta_{4}h\left[x_{9},\;K_{2}\right]+\zeta_{1}h\left[x_{8},K_{2}\right]\right),\;1\right\}$
c_1_	C1	$\frac{dc_{1}}{dt}\text{= }qx_{3}-\lambda h\left[c_{1},K_{2}\right]x_{4}-\eta c_{1}$
c_2_	C2	$\frac{dc_{2}}{dt}\text{= }qx_{4}-\lambda h\left[c_{2},K_{2}\right]x_{3}-\eta c_{2}$
c_3_	C3	$\frac{dc_{3}}{dt}\text{= }qx_{6}-\lambda h\left[c_{3},K_{2}\right]x_{7}-\eta c_{3}$
c_4_	C4	$\frac{dc_{4}}{dt}\text{= }qx_{7}-\lambda h\left[c_{4},K_{2}\right]x_{6}-\eta c_{4}$

$h\left[x,K\right]\left(t\right)\;\equiv \frac{x{\left(t\right)}^{3}}{K^{3}+x{\left(t\right)}^{3}\;}$ denotes a Hill function with half-saturation constant K. $1{\left(t\right)}_{a\leq t\leq b}\equiv \{\begin{array}{c} 1,\;for\text{ a}\leq t\leq b\\ 0,\;else \end{array}$ is the indicator function. R[β,n] describes a linear chain corresponding to an Erlang distribution with rate parameter β and shape parameter *n*.

Model simulations were conducted with poly(I:C)-induced stimulation of IFN-I production in a time-frame of 4 weeks, and kinetic parameters were chosen in line with reported orders of magnitude for processes such as cell-proliferation and cell-differentiation (27) (**Table 2**; **Supplementary Table 2**). To be in line with animal experiments that were stopped before onset of severe disease, we ran simulations only up to 95% of maximal damage levels and defined that point as end-stage renal disease (ESRD), which is used as standard for normalization (**Supplementary Figure 3A**). Model simulations were initialized with all effector cell populations set to zero, and simulations evolved slightly differently between WT and NZB/W conditions already before poly(I:C) supply (**Supplementary Figure 3B**).

**Table 2:** Selected model parameter values.

Param	Description	Value	Unit	Comment
γ	maximal cell proliferation rate	2.4	d^-1^	Mean proliferation time ~10 h; BNID 111159, 101560
δ	cell removal rate	0.24	d^-1^	Mean cell lifetime ~4 d; BNID 101940
β	maximal cell infiltration rate	2.4	d^-1^	Matches proliferation time scale (~10 h)
α	cell activation rate	4.8	d^-1^	Estimated based on ~5 h time-scale of transcription
λ	maximal cytokine endocytosis rate	1.44×10^1^	d^-1^	uptake time ~10 min; BNID 112725
q	maximal cytokine secretion rate	1.44×10^0^	d^-1^	Secretion time 0.6 h^−1^; BNID 112718
η	Cytokine degradation rate	0.24	d^-1^	Degradation time scale ~4 d; Ref (27).

BNID, Bionumbers ID number.

To analyze the model, we first turned our attention to IFN-I kinetics. Poly(I:C) injections initially lead to a first peak in IFN-I concentration between week 19–23 under both NZB/W and WT conditions (**Figure 3B**), followed by decay to base-level under WT and to a damage-associated secondary peak in IFN-I concentrations at 25–27 weeks under NZB/W conditions, in line with experimental data (**Table 3**). In the model, that secondary increase in IFN-I levels after removal of external IFN-I supply via poly(I:C) is caused by self-amplification of the system with formation of immune complexes in the lupus-prone genetic background of NZB/W mice (cf. **Figure 3B**), and is accompanied by increasing amounts of immune-complex deposition as well as MOMA, MO, vNK and tILC populations as well as cytokine concentrations (**Figure 3C**; **Supplementary Figure 3C**). Simulations under wild-type (WT) conditions, that is in the absence of autoantibody-mediated immune complex deposition, generated a transient increase in tissue damage showing recovery to baseline after poly(I:C) removal.

**Table 3:** Published results on NZB/W mice and comparison to model simulations.

Condition	Measure	Observation	Simulation
WT mouse + poly(I:C)	Single-peak IFN-I response	Peak at week 19 (Ref. 2)	Peak at 19–23 weeks (**Figure 3B**)
NZB/W + poly(I:C)	Two-peak IFN-I response	First peak at week 19, second peak at week 25 (Ref. 2)	First peak at 19–23 weeks, second peak at 25–27 weeks (**Figure 3B**)
NZB/W untreated	Death from ESRD	8 weeks after onset of proteinuria (Ref. 18)	Pre-ESRD reached ~7.6 weeks before ESRD (**Supplementary Figure 5B**)
NZB/W + poly(I:C)	Onset of proteinuria	after 25 weeks in 96% of mice (**Figure 1B**)	after 25 weeks ~92% development of pre-ESRD (**Figure 5G**) ^1^
NZB/W + aAGM1	Onset of proteinuria	after 25 weeks in 23% of mice (**Figure 1B**)	after 25 weeks ~26% development of pre-ESRD (**Figure 5G**, ‘tILC depletion’) ^1^
NZB/W untreated	Onset of proteinuria	20–44 weeks, median 40 weeks (Ref. 2); 13% of mice after 25 weeks (**Figure 1B**)	after 25 weeks ~15% development of pre-ESRD (**Figure 5G**, ‘hybrid’) ^1^

^1^Estimates based on the observed delay of 8 weeks after onset of proteinuria reported from untreated NZB/W mice.

Next, we analyzed the influence of the five feedback mechanisms included in our model on the damage value (**Figure 3D**; **Supplementary Figure 3D**). Removing individual feedback loops could be compensated by other branches in the model to a large extent, although eliminating F3 and especially F5 alone already had a substantial impact on model dynamics. Removing the combination of immune-complex mediated feedback on both the tissue and vessel compartments, that is F3/5, reduced damage levels almost to the wild-type condition, which is equivalent to F3/4/5. Additional elimination of direct damage-induced IFN-I release further reduced tissue damage in all those cases (F1/3, F1/5, F1/3/5), recapitulating the importance of elevated IFN-I levels for driving severe disease progression observed in the clinics (11–13, 15). Finally, regarding sensitivity to cell-state dynamics in the model, we found that a reduction of the rates of immune-cell infiltration or increasing death rates would prevent the development of ESRD (**Figure 3E**; **Supplementary Figure 3E**), and vice versa, elevated immune-complex levels would promote rapid ESRD onset, according to model simulations.

Taken together, our analysis using mathematical modeling shows how auto-amplification cycles amongst innate immune-cell populations, in combination with damage-associated IFN-I release, can determine onset of chronic autoimmune disease in individuals that have genetic predisposition for immune-complex deposition.

### 2.3 Spontaneous inflammatory events can induce heterogeneous, advanced onset times of chronic inflammation

**Figure 4:**
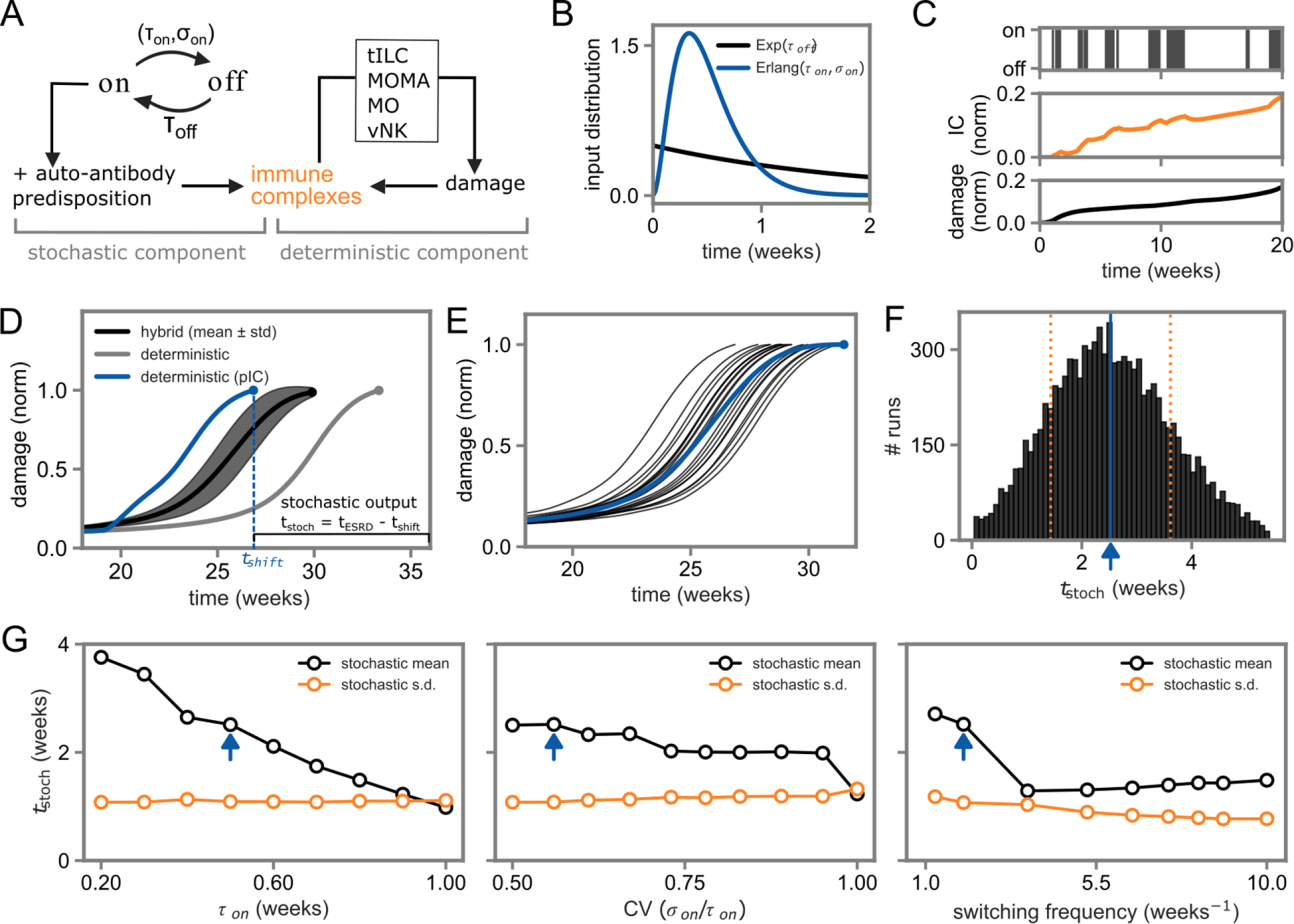
Model simulations of stochastic disease-onset dynamics in the presence of spontaneous inflammatory events. **(A, B)** Schematic and input distributions of the hybrid stochastic and deterministic model. The stochastic model alternates between on- and off-states of acute inflammatory events, where immune-complex production occurs only in the on-state, feeding into the deterministic model of cell-cell interactions shown in **Figure 3A**. **(C)** Representative model simulation showing the stochastic inflammatory process and the resulting dynamics of immune-complex deposits and tissue damage. **(D)** Damage kinetics for the hybrid model as well as the deterministic model with and without poly(I:C) treatment. The black line and shaded region represent average and standard deviation based on 100 simulations. The stochastic output t_stoch_ is defined as time to ESRD in the hybrid model after subtracting the time to ESRD under poly(I:C) treatment. **(E)** Tissue-damage kinetics for 20 stochastic kinetics of spontaneous inflammatory events and corresponding average kinetics (blue). **(F)** Histograms of the stochastic output t_stoch_ as shown in panel **(D)** based on 10000 stochastic simulations. The blue line indicates the mean of the distribution and the orange dotted lines denote the standard deviation. **(G)** Analysis of the stochastic process. Average and standard deviation of the stochastic output t_stoch_ are shown for varying values of the average duration τ_on_, coefficient of variation (CV) and switching frequency of inflammatory events. Blue arrows indicate default parameter values as in panel F.

Due to their predisposition to immune complex deposits, NZB/W mice develop lupus nephritis even in the absence of poly(I:C) treatment. The onset time of lupus nephritis is highly heterogeneous in the time period between 20–44 weeks of age, averaging at around 37 weeks (**Supplementary Figure 4A**). To investigate this phenomenon, we formulated a hybrid model by adding a series of acute inflammatory events described by a telegrapher’s process, that is switching between on- and off-states after stochastic time-intervals with average *τ_on_, τ_off_* (**Figure 4A**). To account for the complex multi-step process in removing of acute inflammatory events and recovering to base-line, which is not explicitly represented in the model, we described the on-state period using an Erlang distribution with variable standard deviation *σ_on_*, (**Figure 4B**), in line with our response-time modeling framework and other modeling studies (25, 28, 39–41). Immune-complex deposits are increased in the presence of spontaneous inflammatory events, so that immune complexes can accumulate if by chance higher levels of immune complexes arise than can be effectively degraded.

Indeed, we found that this mechanism, coupled to our model of disease-onset driven by ILC-mediated positive-feedback loops (cf. **Figure 3A**), resulted in spontaneous increase of immune-complex deposits followed by acquisition of tissue damage (**Figure 4C**; **Supplementary Figure 4B**). While the deterministic model without poly(I:C) as strong initial stimulus alone was already able to explain delayed damage acquisition in that scenario, the hybrid model fell in between the deterministic models with and without poly(I:C) regarding disease kinetics and thus reflected earlier disease-onset due to environmental conditions or acute inflammatory events (**Figure 4D**). Importantly, the hybrid model accounted for the observed strong heterogeneity in disease-onset kinetics (**Figures 4E, F**). While increasing the total on-time of the stochastic process induced a shift to faster disease-onset at nearly constant heterogeneity in terms of the standard deviation, modulating the coefficient of variation of the stochastic process affected both speed and heterogeneity (**Figure 4G**; **Supplementary Figures 4C–E**). Modulating the switching frequency at unchanged total on-time and CV indicated a stochastic resonance phenomenon (42) under periodic forcing, that is the shortest mean-time to ESRD occurs for intermediate values of the switching frequency.

Taken together, our simulations indicate that a series of spontaneously occurring inflammatory events coupled to strong feedback-mediated amplification mechanisms can explain the heterogeneity in occurrence and onset-time of chronic autoimmune conditions.

### 2.4 Effective immunotherapy by ILC depletion shows a strong dependence on treatment onset and duration

**Figure 5:**
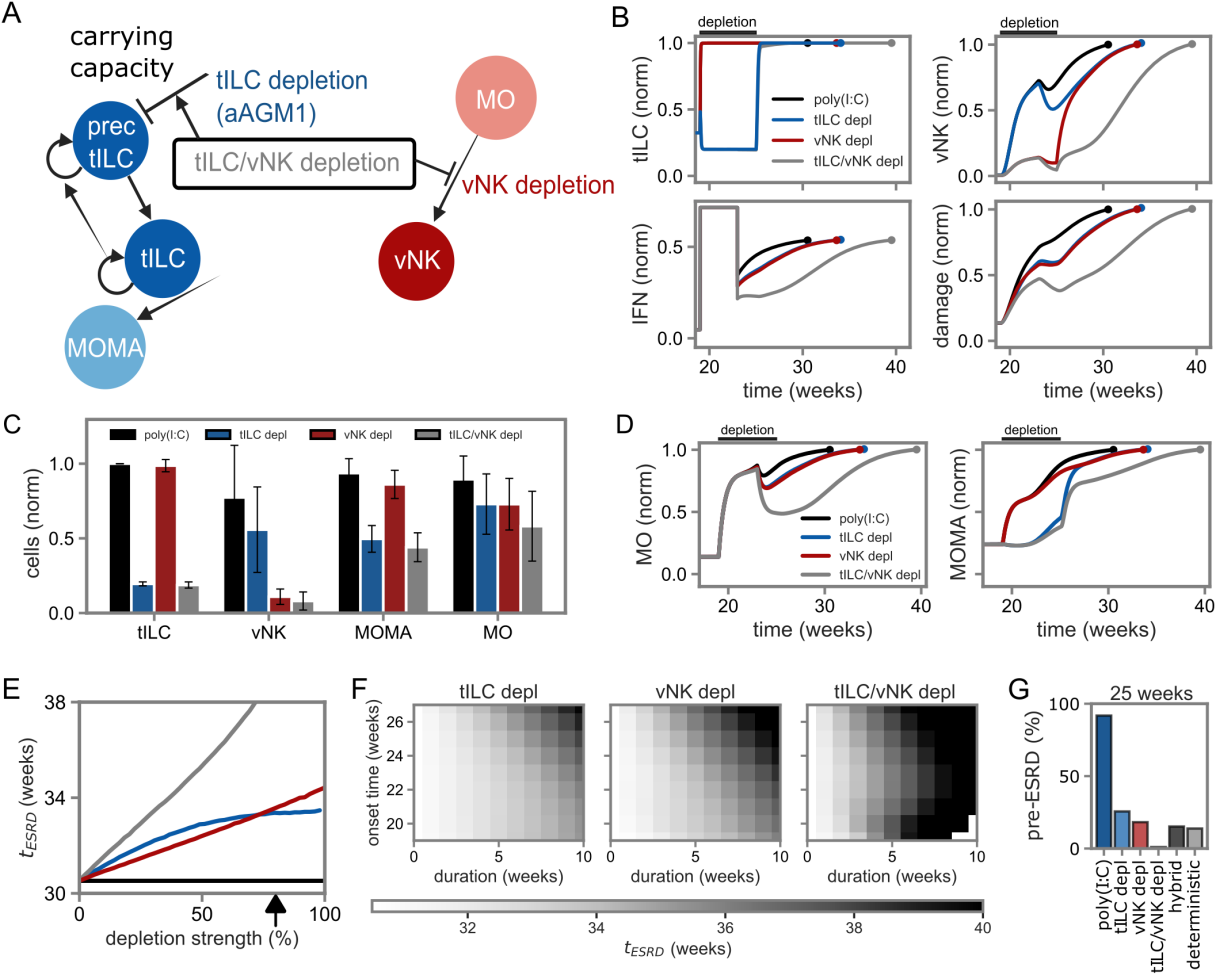
Analysis of NKp46+ ILC depletion in mathematical model simulations. **(A)** Schematic representation of the simulated depletion experiment. tILC depletion corresponds to aAGM1 treatment in experiments (cf. **Figure 1A, B**), in addition we studied vNK cell depletion and combined tILC and vNK cell depletion. **(B–D)** Simulated kinetics and robustness analysis. Simulations are run until ESRD is reached. Bar plots show average and standard deviation of simulations with parameter values sampled from log-normal distributions centered at standard values. **(E)** Time to ESRD for varying depletion strength. The black error indicates the default depletion strength. The y-axis shows the time to onset of ESRD. **(F)** Combined analysis of depletion onset-time and duration on the time to onset of ESRD. **(G)** Percentage of simulations that developed a pre-ESRD after 25 weeks defined as 70% of the ESRD damage value. Percentages are calculated from a robustness analysis for 1000 parameter sets sampled from log-normal distributions centered at standard values. The labeling follows conventions used in **Figure 4D** and **Figure 5C**.

Since previous results emphasized the importance of IFN-I mediated activation of tILC for rapid activation of the inflammatory response (20), we incorporated temporary depletion of tILC and for comparison vNK cells into our model (**Figure 5A**). In the model, depletion was implemented as downregulation of tILC carrying capacities and NK cell migration. Depletion of only tILC reflects antibody-based NKp46+ ILC depletion experiments using aAGM1 antibodies (cf. **Figures 1A, B**). In line with the experimental setup, we chose a depletion period of six weeks starting from the beginning of poly(I:C) injections, that is in the time interval between 19 to 25 weeks of mouse age, at a depletion strength of 80%. While our experimental results supported a strong role of tILC for onset of disease, our systematic analysis indicated that direct feedback on MOMA and MO (F3/5) would dominate the dynamics when focusing on interaction topology at uniform feedback strength for all pathways (cf. **Figure 3D**, F3/5). Hence, for analysis of depletion scenarios, we refined the mathematical model (cf. **Figure 3A**) by down-scaling those feedback strengths accordingly.

Indeed, our depletion setup yielded effective temporary down-regulation of tILC and vNK cells, inducing a substantial delay in damage kinetics and immune-complex deposition (**Figure 5B**). Accordingly, simulations revealed temporary reduction in cell numbers and delayed kinetics in MOMA and MO as well as cytokine concentrations upon depletion (**Figures 5C, D**, **Supplementary Figure 5A**), in line with the role of vNK cells and tILC as amplifiers of inflammatory processes at the onset of lupus nephritis. Depleting both vNK cells and tILC caused a stronger reduction of cell counts in both the MOMA and MO compartments, indicating interaction across model compartments. The reduced cell counts under depletion conditions resulted in decreased damage values in the time-interval of depletion, which recovered to ESRD by week 35 except for the case of combined vNK cell and tILC depletion (**Figure 5D**). Interestingly, for increasing depletion strengths, the model predicts a saturating dependence of ESRD onset time in the case of tILC depletion, a linear dependence for vNK cell depletion, and a super-linear dependence in case of the combined tILC and vNK cell depletion (**Figure 5E**). A combined analysis of depletion time and duration (**Figure 5F**) revealed that longer treatment durations initiated concurrently with poly(I:C) stimulation are most effective, while after disease-onset, depletion no longer influenced ESRD onset and did not prevent disease progression. Finally, to compare our model to proteinuria onset in mice, we defined pre-ESRD at 25 weeks as reaching 70% of the ESRD damage value, in line with a reported time-span of 8 weeks to reach ESRD upon proteinuria in untreated NZB/W mice (18)(**Supplementary Figure 5B**; **Table 3**). We performed simulations with parameters sampled from log-normal distributions around standard values, recapitulating experimental observations (**Figure 5G**; **Table 3**, cf. **Figure 1B**). In particular, almost all poly(I:C) simulation runs reached pre-ESRD, and simulation runs under condition of tILC depletion, corresponding to aAGM1 treatment, developed pre-ESRD in ~26% of simulation runs. Simulations of our hybrid deterministic and stochastic model capturing untreated NZB/W mouse conditions resulted in ~15% pre-ESRD, compared to 13% in our experimental system.

Overall, the model predicted delayed or impaired disease-onset by tILC and vNK cell depletion in a synergistic manner, and recapitulated disease-kinetics observed in our *in vivo* experimental system.

## 3 Discussion

By analyzing single-cell RNA sequencing data, we found that inflammatory processes in lupus nephritis are associated with increased cell numbers of activated tILC and vNK cells, which promote the expansion of disease-associated macrophages and exacerbate epithelial cell injury (20). Hence, an overall increased activation of innate immune cells precedes chronic inflammation. Based on that finding, we developed a conceptual modeling framework and a specific mathematical formulation of immune-cell interactions at the onset of lupus nephritis.

Our data analyses revealed that NK cell and helper ILC activation is a dynamic, multi-dimensional process that occurs within each population but can lead to distinct functional outcomes, including cytotoxicity, cytokine production, and immune regulation. Overall, transcriptional dynamics show increased numbers and a pronounced differentiation trajectory of activated tissue-associated ILC and vessel-associated NK cells in nephritic mice. These results highlighted how a common activation process can diversify into specialized effector programs depending on the cell type, activation trajectory, and tissue context, and they set the stage for a mathematical model formulation based on activated vNK cell and tILC compartments as major drivers of disease.

In model development, we derived a flexible mathematical framework that allows mathematical formulation and systematic analysis of the involved cell-cell interaction systems that are at the core of immunological control circuits. Models are almost entirely formulated in terms of dimension-free variables and take only a minimal set of parameters for processes such as cell proliferation and activation as input, which we assigned to plausible orders of magnitude. The modelling framework can be adapted to other systems exhibiting cell-cell communication and cell-proliferation dynamics in future research.

Our specific model implementation reconciles the recent observation of ILC as an important driver of chronic inflammation with established knowledge on the pathophysiology of SLE, such as the presence of autoantibodies in lupus-prone individuals that is likely associated with genetic predisposition, and the important role of IFN-I for disease progression. Structurally, the model is composed of a course-of-events of IFN-I induction followed by vNK cell and tILC induced recruitment of monocytes and macrophages, and their combined action to provoke tissue damage, which leads to a sustained, chronic inflammatory state in the presence of immune complexes, thus closing a positive feedback loop. Regarding system dynamics, our model hypothesis is a rapid disease progression in the case of NZB/W conditions and poly(I:C) treatment, which is prevented in WT mice and delayed by vNK cell or tILC depletion.

We were wondering whether the outlined amplification mechanism mediated by innate immune cells may provide a plausible explanation for the heterogeneity in timing and occurrence of disease onset in lupus-prone individuals, which is also a hallmark of the NZB/W F1 system. That hypothesis was motivated by the well-known characteristic of positive feedback driven amplification systems to facilitate all-or-none decisions in a noisy environment, such that instead of graded disease levels, the expected outcome would stabilize disease progression once a certain threshold level in inflammation intensity is reached. As a source of noise, we here assumed a stochastic series of acute inflammatory events that may or may not be physiologically recognizable as small to intermediate level infections or autoimmune reactions in humans or animals. Alternatively, one could also think of other environmental stimuli as potential noisy triggers of disease onset, either directly via IFN-I stimulation or also indirectly via other processes mediating local tissue damage or modulating immune cells. Indeed, our stochastic model simulations were in good agreement with published time-course data on NZB/W mice without poly(I:C) treatment.

Previous mathematical modelling studies on autoimmune diseases have focused on the recurrent, oscillatory dynamics of acute flares in an established chronic disease (43, 44), or on T cells and their interactions and spatio-temporal dynamics (44, 45). Moreover, several mathematical models were developed to describe mechanisms of autoimmune disease, often attributed to chronification mediated by positive feedback loops within the immune response (24, 44, 46). Our model builds on these approaches by explicitly incorporating feedback via immune complexes and type I interferon signaling, and combines it with another network motif governing tissue homeostasis which is known to describe bistable system dynamics. The combination of these motifs gives rise to a network structure that captures autoimmune disease behavior more broadly. Notably, we demonstrated that the same parameter set can reflect both, chronic disease in individuals with genetic predisposition and effective tissue repair in others. Furthermore, the framework incorporates a hybrid model formulation including stochastic input signals capturing disease heterogeneity.

This study aimed to elucidate the mechanistic origins of chronic inflammation based on biologically plausible assumptions, rather than striving for predictive accuracy. In complex systems such as immune responses marked by diverse, interdependent components, mechanistic modelling can generate testable hypotheses and identify key regulatory pathways (1, 5, 47). That approach is particularly valuable in autoimmune diseases, where patient heterogeneity and non-linear immune cell dynamics as well as the lack of experimental data with high time resolution hamper the data-annotation of detailed mechanistic models. By focusing on causal relationships, we explored the conditions that enable or prevent disease chronification, and the conditions for effective treatment by means of ILC depletion. In line with previous work indicating a window-of-opportunity for effective perturbation at the onset of T-cell mediated chronic inflammation (25), our simulations revealed a strong dependence of effective depletion therapy on treatment onset time.

While our findings provide mechanistic insights, it is important to acknowledge the limitations of the NZB/W F1 mouse model, which may not fully capture the complexity of human SLE, including genetic diversity and disease heterogeneity (48, 49). Consequently, translation of our results to human disease should be approached cautiously, and further work integrating patient-derived data will be essential to validate and refine model predictions. Rather, our study highlights general principles that may set the stage for advanced experimental and therapeutic strategies in SLE.

Collectively, our findings support the existence of a positive-feedback based amplification mechanism, contributing to the initiation and maintenance of chronic disease. Our model captures both the observed high variability in damage kinetics and delayed or impaired onset of chronic disease under aAGM1 treatment mediating ILC depletion in tissue.

## 4 Materials and methods

### 4.1 Data sets and mice

To investigate transcriptional dynamics, we reanalyzed publicly available single-cell RNA sequencing kidney data from Biniaris-Georgallis et al. (20), focusing on kidney samples from NZB/W mice (control: GEO GSM6280142, disease: GEO GSM6280144). The dataset comprises 6,032 cells from 10 young mice (12 weeks old, pre-treatment) and 8,723 cells from 8 nephritic mice (25 weeks old, post-poly(I:C) treatment). Additionally, we obtained single-cell RNA sequencing data on vessel-associated ILC populations from NZB/W mice. Following the experimental approach described in Biniaris-Georgallis et al. (20), CD45+ kidney cells were further enriched for vessel-associated cells (CD45.2i.v+) and harvested from 10 mice prior and 6 mice after poly(I:C) treatment. In particular, mice received intraperitoneal injections of 200 µg poly(I:C) three times per week for four weeks, starting at 19 weeks of age. For tILC depletion, twenty microliters of reconstituted rabbit anti-mouse anti-asialoGM1 antibody (Fujifilm Wako, 014-09801) were diluted in PBS to a final volume of 200 µL per injection. All mice were kept in the FEM animal facility of Charité-Universitätsmedizin Berlin under standard pathogen-free conditions. All animal experiments were approved and were in accordance with the guidelines of the local animal care and use committees (Landesamt für Gesundheit und Soziales, Berlin). Furthermore, we reanalyzed a publicly available human single-cell RNA sequencing dataset of kidney leukocytes from patients with lupus nephritis reported by Arazi et al. (50). The dataset comprises renal biopsy samples from 24 patients with lupus nephritis and 10 healthy kidney donors serving as controls. The cohort includes predominantly female patients typical for lupus nephritis, with adult subjects across a broad age range.

### 4.2 Data analysis

The sequencing data were mapped to the mouse reference genome (mm10 pre-built references v.3.0.0) provided by 10x Genomics using the CellRanger suite (v.3.0.1). Count matrices were generated by CellRanger and used for the further analysis. Assessment of mapping quality was carried out using the CellRanger summary statistics. Unless otherwise specified, the analysis was performed using the Seurat pipeline (version 5.1.0). Both the tissue-associated and vessel datasets, as well as the nephritic and young samples, were processed independently following the filtering criteria from Biniaris-Georgallis et al. (20). Briefly, quality control was performed separately for each dataset based on the number of detected genes, total UMI counts, and mitochondrial transcript content. Cells with fewer than 500 or more than 6,000 detected genes or with >75% mitochondrial reads were excluded, and genes expressed in fewer than three cells were removed prior to downstream analyses. After preprocessing, the murine datasets were integrated based on 2,000 highly variable features. Prior to integration, filtered datasets were log-normalized, highly variable genes were identified using the variance-stabilizing transformation (2,000 features), and expression values were scaled before principal component analysis. The number of principal components used for downstream analyses was selected based on inspection of elbow plots. Initial low-dimensional embeddings indicated partial clustering by sample origin, consistent with the presence of batch effects. To correct for these effects, datasets were integrated using Seurat’s anchor-based integration framework. Integration quality was assessed by improved mixing of cells across samples, reduction of dominant sample-specific clustering, and preservation of expected lineage-specific marker gene expression. Cell clustering was performed using the first 20 principal components and the Louvain algorithm with a resolution parameter of 0.9, identifying 19 distinct clusters, which were annotated using marker genes from the original publication. A general cell type annotation was performed using canonical lineage markers. NK cells were defined by their expression of *Eomes*, while hILCs were identified based on general *Il7r* expression. To ensure accurate cell type assignment, we excluded cell clusters with lacking or mixed NK and hILC marker gene expression patterns based on the genes shown in **Figure 1D**. We furthermore excluded 16 cells that were embedded distantly from the main NK and ILC clusters and in close proximity to the “unknown” cell populations in the UMAP space, suggesting ambiguous transcriptional profiles. One cluster exhibited high expression of C1qc, *Csf1r*, *Apoe*, and *Trem2*, and was annotated as a macrophage population. To further characterize the clusters, we calculated signature genes for each cluster, compared to all others combined, using the FindMarkers function (**Supplementary Table 1**). To specifically check for cytokines, we filtered the signature genes using the gene list published by Stubbington et al. (51) for genes with an FDR of less than 10% and a log2-foldchange of at least 0.25 which were expressed in at least 1.5% of cells, in both contrasts. To enable clearer comparisons, gene expression values were normalized per cytokine across all anatomical sites and disease conditions, such that the highest expression value for each gene was scaled to 1. To further characterize the identified cell types, we filtered the signature genes using a false discovery rate (FDR) threshold of 10% and ranked them by log2-fold change. For each cluster, the top five signature genes were selected based on log2-fold change. Cellular activation states were assessed for NK cells and hILCs using gene expression-based metrics. NK cell activation was determined using the murine KEGG’s “Natural killer cell mediated cytotoxicity” gene set, while hILC activation was evaluated using the activation signature defined by Biniaris-Georgallis et al. (20). Both sets were filtered for genes which were expressed in at least 10% of the cells. The activation signature score was calculated using the AddModuleScore function from Seurat. We filtered for the 95% interval and the scores were scaled between zero and one. To account for differences in sample sizes, the datasets were downsampled to match the young sample size within the vessel. Developmental trajectories were computed using monocle3 (version 1.0.0) using 5 nearest neighbors. Pseudotime was initialized using the highly proliferative Mki67^++^ vNK and the tissue-resident Zfp683^+^ tILC population as roots (“S”). For investigation of relative cell-population sizes using down-sampling, we repeatedly subsampled the whole single-cell data set 1,000 times and computed the standard deviation of the NK and ILC percentages. A permutation test was performed on the down-sampled single-cell dataset by randomly shuffling control and disease labels (n = 10,000) within tissue and capillary compartments separately, to assess whether observed differences in cell type abundances across conditions could be attributed to random variation.

Further, we reanalyzed a published human single-cell RNA-seq dataset of lupus nephritis (Arazi et al. (50)), which was processed as previously described by Biniaris-Georgallis et al. (20). In brief, cells were filtered for counts (nCount_RNA< 600,000) and genes (nFeature_RNA< 6,000). Dimensionality reduction, clustering and analysis of DEGs was performed following read count normalization and log-transformation, the top 2,000 highly variable genes were selected. Cells were annotated according to the cell type definitions reported by Biniaris-Georgallis et al., based on canonical lineage marker expression. ILCs were further subset and reclustered independently to resolve subpopulations, resulting in two distinct hILC clusters (resolution parameter 1.0 using 15 dimensions). To assess activation states, NK cell activation was scored using the human KEGG “Natural Killer Cell Mediated Cytotoxicity” gene set, filtered for genes expressed in at least 10% of cells. ILC activation was evaluated using the human orthologs of the murine activation signature employed in the main analysis, applying the same filtering criteria. Module scores were calculated using Seurat’s AddModuleScore function, restricted to the 95%. For visualization of selected genes, expression values were scaled (z-score) within each cell type compartment to enable comparison across subclusters.

### 4.3 Mathematical models

Based on the modelling framework described in Section Results (**Figure 2** and Equations), we formulated a specific mathematical model (**Table 1**) in which cell-state dynamics are governed by four core processes each governed by a single rate parameter: activation (α), infiltration (*β*), proliferation (*γ*), and decay (*δ*). The IFN-I concentration c (t) is described using a quasi-steady-state assumption, reflecting the faster cytokine kinetics relative to cellular dynamics, and is determined by three concentration levels: baseline concentration (*q_0_)*, poly(I:C) induced concentration (*q_1_)*, and damage and immune complexes induced concentration (*q_2_)*. To capture homeostatic regulation of monocytes and macrophage-derived monocytes, network interaction with cEC and iPEC with carrying capacities (ω) are considered which are determined by cytokine dynamics including production (*q*), endocytosis (λ), and decay (η). Consistent with the experimental system, we chose a time-scale of 6 weeks (*µ_1_*) for damage acquisition. Genetic predisposition for the presence of autoantibodies in lupus-prone individuals is reflected by damage-associated immune-complex deposition (*µ_2_*). The complete set of model parameters used for simulations are listed in **Table 2** and **Supplementary Table 2**.

#### 4.3.1 tILC and vNK cell depletion

To account for the NKp46+ ILC depletion experiment using aAGM1, a control variable κ∈[0,1] is introduced, accounting for downregulation of tILC carrying capacities as well as NK cell migration. We have chosen a depletion strength of κ=0.8, consistent with reports that spontaneous NK cell cytotoxic activity can be reduced by more than 80% relative to controls (52). Furthermore, we have chosen the feedback parameters $\zeta_{3}=\zeta_{5}=0.2$ downregulating the migration of MOMA and MO due to immune complex recognition to emphasize the importance of NKp46+ ILC in the model topology. The model equations for tILC and vNK cells are modified as follows:


$$\begin{array}{l} \frac{dx_{1}}{dt}\text{= }\gamma \left(1-\frac{\left(x_{1}+x_{2}\right)}{\omega \kappa }\right)\left(x_{1}+x_{2}\right)-\\ \alpha min\left\{h\left[c,K_{1}\right]+\zeta_{2}h\left[x_{8},K_{2}\right],\;1\right\}x_{1}-\delta x_{1} \end{array}$$

$$\frac{dx_{5}}{dt}\text{= }\beta h\left[x_{6},K_{3}\right]\kappa -\delta x_{5}$$

#### 4.3.2 Stochastic simulation of acute inflammatory events

To describe acute inflammatory events, we extended the deterministic framework by incorporating a stochastic telegrapher’s process switching between an off-state ( $X_{off}$) and an on-state ( $X_{on})$, each taking the values 0 or 1. The process is governed by the two reversible reactions $R_{1}:\;X_{off}\to X_{on}$ and $R_{2}:\;X_{on}\to X_{off}$. Here, reaction $R_{1}$ follows a Poisson process with rate parameter *k_off_*, and $R_{2}$ is modelled using our response-time framework (25, 28) (see below) with distribution $R\left[k_{on},n\right]$ to account for delay in recovering to the uninflamed state. The stochastic dynamics generated by this process are integrated into the ODE model above by modifying the equation for immune complexes as follows:


$$\frac{dx_{9}}{dt}\text{= }\mu_{2}\left(\xi X_{on}+y_{3}\right)-\nu_{2}x_{9}$$

Here, ξ∈(0,1) is a scaling factor adjusting immune complex production by means of acute inflammatory events.

#### 4.3.3 Response-time modelling

To incorporate delays into our models, we use a response-time modelling framework based on the linear chain trick (25, 28, 53). This approach approximated the effect of omitted intermediate processes, such as immune complex production by B cells, by introducing time delays through systems of irreversible multistep ODE:


$$\frac{dy_{1}}{dt}\text{=- }ky_{1},\;\frac{dy_{i}}{dt}\text{=- }k(y_{i-1}-y_{i}),\;\;\frac{dy_{n}}{dt}\text{= }ky_{n}$$

for i=2, …n. This ODE system introduces a delay distribution according to an Erlang distribution with mean τ=n/k and standard deviation $\sigma =\sqrt{n}/k$. The Erlang distribution is a special case of a Gamma distribution with integer shape parameter. We denote the multistep ODE chain by R[k,n], where *k* denotes the rate constant and *n* the number of sequential steps.

### 4.4 Software and simulations

All ODE models are solved numerically using the *lsoda* method from the SciPy library switching automatically between the non-stiff Adams method and the stiff BDF method. The stochastic acute inflammation model is simulated using the stochastic simulation algorithm (SSA) also known as Gillespie’s algorithm with initial condition $X_{off}=1$ and $X_{on}=0$. Before analysis, we normalized model kinetics using an end-stage renal disease as reference, which is the time-point where 95% of the maximal damage in the NZB/W mice with poly(I:C) treatment condition is reached. For robustness analysis, each parameter is sampled from the log-normal distribution with zero mean and standard deviation set to 10% of the default parameter value, and N=1000 simulations with different parameter sets are performed. Sensitivity analysis is conducted by keeping all model parameters fixed at their default values while varying one individual parameter by 20% up or down.

## Data Availability

The datasets presented in this study can be found in online repositories. The names of the repository/repositories and accession number(s) can be found below: https://www.ebi.ac.uk/arrayexpress/, E-MTAB-16156. The code for the single-cell analysis and the mathematical modeling can be accessed through https://gitlab.com/t8145/thurleylab-publications/lupus.
