# Supplementary material for: Amplification cycles through innate lymphoid cells at the onset of lupus nephritis
Source: Front Immunol. 2026 Mar 12;17:1756560. doi: 10.3389/fimmu.2026.1756560 (PMC13018116; doi:10.3389/fimmu.2026.1756560)
Supplement: Supplementary file 1 [file Supplementaryfile1.pdf]

Supplementary Material

1 Supplementary Text: Detailed model description

1.1 Specific cell-cell interaction model

The following processes were considered in model formulation (cf. Figure 3A): (i) injection of poly(I:C) leads to increased IFN-I concentrations (1,2). (ii) IFN-I activates tILC in the kidney tissue (1,3,4) and monocytes in the capillaries. (iii) tILC are an important source of cytokine-mediated growth control for myeloid cells, for example through IFN-γ, TNFα and GM-CSF, and thus cause immigration and proliferation of monocyte-derived macrophages (MOMAs) (5) leading to further tissue damage (2). Next, (iv) MOMAs produce various pro-fibrotic growth factors like IL1-β, TNFα and CCL2, activating intermediate parietal epithelial cells (iPECs) in Bowman's capsule (1). In addition, iPECs produce cytokines like CCL2, CCL5 and IL-34 that stimulate MOMAs to proliferate (1), thereby mutually amplifying the growth rates of both iPEC and MOMAs. A similar network module is formed in the capillaries (v) by activated monocytes and capillary endothelial cells (cEC), occurring in increased number in nephritic mice (1) and are therefore chosen as key regulators in the homeostasis model in the capillary compartment. Increasing activated monocyte numbers lead to NK cells infiltration to the vessel compartment, having a strong cytotoxic capacity and directly cause tissue damage upon activation (6). Tissue damage induced by vNK cells, MOMA and activated monocytes creates a positive feedback loop that prolongs and chronifies the inflammatory process. (vi) Tissue damage increases type I IFN concentrations by increased damage and immune complexes (7,8), which leads to increased activation of tILC and monocytes.

Genetic pre-disposition for the presence of autoantibodies in lupus-prone individuals is reflected by (vii) damage-associated immune-complex deposits (9,10), leading to additional recruitment of monocytes (11) and activation of MOMA (5), thus closing the loop to tissue damage (12,13). Immune-complex accumulation incorporates a delay $R[\beta, n]$ represented in terms of our response time modelling framework (14,15) (see below), to describe time-lagged onset of immune complex production by plasma cells. $\{x_1(t), \dots, x_7(t)\}$ represent cell densities, $x_8(t)$ and $x_9(t)$ the amount of tissue damage and immune complexes, respectively. The concentration of IFN-I is denoted by $c(t)$, concentrations of cytokines by $\{c_1(t), \dots, c_4(t)\}$, and ζ_i, i=1, …, 5, is the feedback strength of feedback F1 … F5 (cf. Figure 3A). In all simulations, we chose the initial condition $x_1(t)$=1, while all other model states are set to zero.

1.2 Non-dimensionalization and parameter annotation

To work with dimensionless quantities, we represent cells, cytokines, immune complexes, and tissue damage in arbitrary units (a.u.) representing specific scales for each category separately. Cellular proliferation and infiltration are both assumed to occur with a mean duration of 10 hours, while the mean cell lifetime is around 4 days. Cell activation is defined twice as fast as proliferation and infiltration, with a mean activation time of 5 hours. To normalize cell kinetics, we set the carrying capacities of tissue-resident tILC low, iPEC, and cEC to 1 a.u. For IFN-I dynamics, we define three concentration levels: a baseline concentration of 0.05 a.u., a maximal induced concentration of 1 a.u. upon poly(I:C) stimulation, and a damage- or immune complex-induced concentration set to 0.4 a.u. Cellular responses to IFN-I are implemented using Hill-type functions with a half-saturation constant

of 0.4, reflecting the IFN-I level typically generated in response to damage or immune complexes. Cytokine dynamics operate on a faster time scale, measured in minutes. Cells internalize 1 a.u. of cytokines with a mean uptake time of 10 minutes. Production occurs more slowly, with a mean production time of ~1.5 hours, following Adler et al. (21). Degradation occurs on a slower time scale of ~4 days, similar to cellular turnover (21). For immune complexes, we assume the same degradation rate as for cells and cytokines, with a mean removal time of 4 days. Tissue damage decreases with an average removal time of ~8 weeks. Damage production is defined with a mean time of 6 weeks, consistent with mouse data showing lupus nephritis onset ~6 weeks after poly(I:C) treatment. To normalize immune complex kinetics, production and degradation rates are matched. For cytokines, immune complexes, and tissue damage, we apply Hill functions with a half-saturation constant of 1.5, reflecting steady-state concentrations on the order of magnitude one.

2 Supplementary Tables and Figures

Table S1: Supplementary data table, provided as separate file. (**Tab 1**) Expression values of signature genes of major cell types corresponding to Figure S1A. (**Tab 2**) Expression data of curated marker genes displayed in Figure 1D. (**Tab 3**) Expression levels of cytokines as shown in Figure S1B. (**Tab 4**) List of genes used to calculate activation scores for vNK and tILC populations.

Table S2: Supplementary parameter values.

**Param**	**Description**	**Value**	**Unit**	**Comment**
μ_1	maximal growth rate of damage	2.4×10^{-2}	d^{-1}	estimate based on onset of proteinuria after 6 weeks
ν_1	maximal damage removal rate	0.12	d^{-1}	Assumes damage removal time ~8 d
μ_2	immune complex deposition rate	0.24 (NZB/W); 0 (WT)	d^{-1}	Matches IC degradation for kinetic normalization
ν_2	immune complex decay rate	0.24	d^{-1}	Mean IC lifetime ~4 d; aligned with cell and cytokine decay
ω	carrying capacity of cells	1	a.u.	Normalization: capacity set to 1 a.u. for all cell types
$q_{0,}$	baseline IFN-I	0.05	a.u.	Dimensionless baseline IFN-I level
q_1	poly I:C triggered IFN-I	1	a.u.	Normalization: Maximal concentration after poly(I:C)
q_2	damage / IC induced IFN-I	0.4	a.u.	IFN-I concentrations in response to damage or ICs
K_1	half saturation constant IFN-I	0.4	a.u.	Reflects IFN-I level typically induced by damage and IC
K_2	half saturation constant damage, IC, cytokines	1.5	a.u.	Hill threshold ~1 a.u.; reflects typical steady-state values
K_3	half saturation constant monocytes	8	a.u.	reflects order of magnitude of steady-state values; ~10 a.u.
k_{off}	inflammation on-switch rate	0.07	d^{-1}	mean waiting time of ~14 days until the onset of an acute inflammation
k_{on}	inflammation off-switch rate	0.86	d^{-1}	mean duration of ~3.5 days for the acute inflammation
ϑ	Scaling factor MO migration through NK cell activation	0.5	-	reduced MO activation due to IFN-I compared to IC recognition
ξ	IC production factor for acute inflammations	0.1	-	reduced IC production compared to damage induced IC accumulation
n	chain length	3	-	delay control
ζ_i	feedback strength	1	-	Equal contribution of each feedback loop

BNID: Bionumbers ID number.

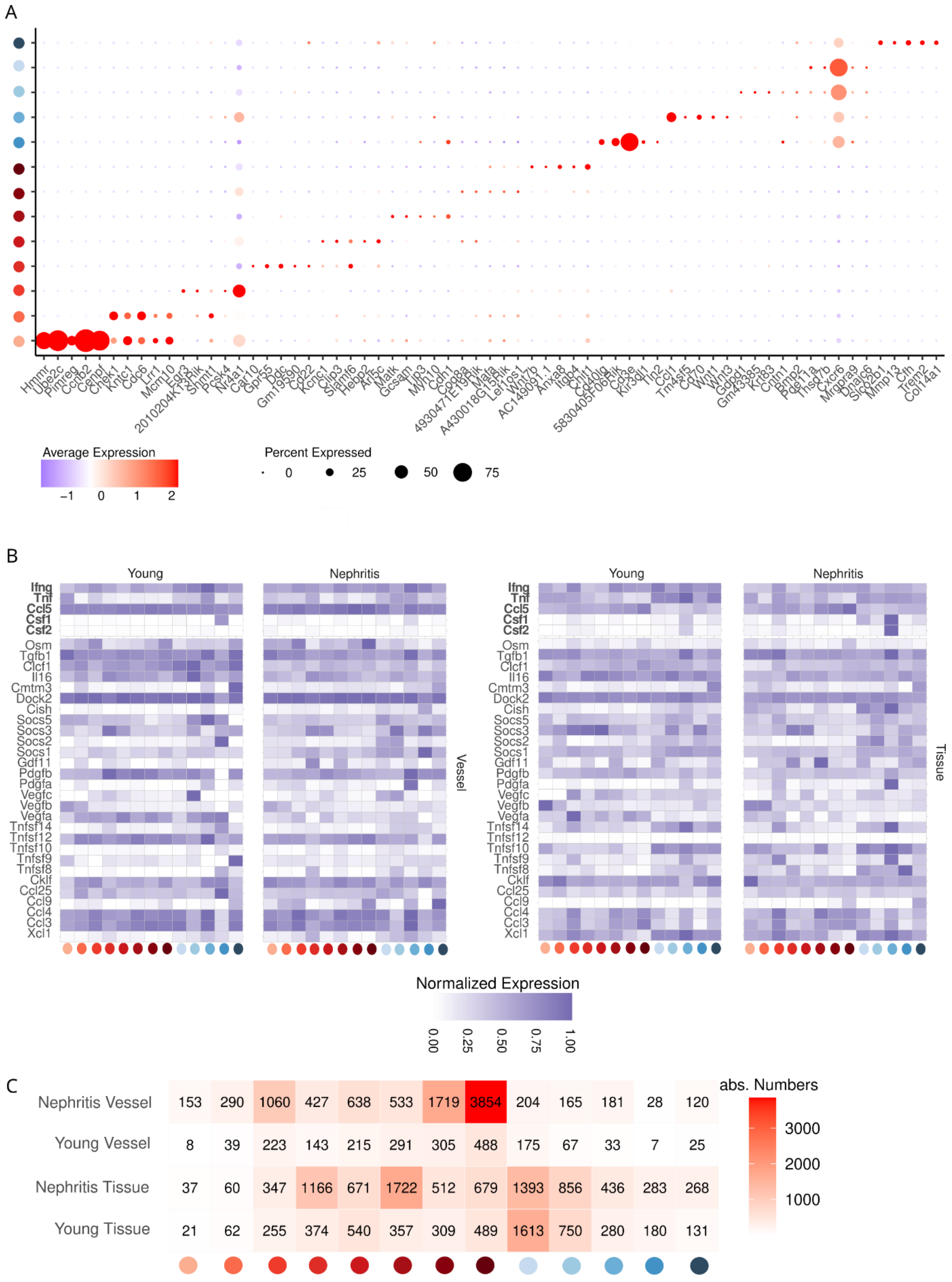
A
Average Expression
−1 0 1 2
Percent Expressed
0 25 50 75
B
Young
Nephritis
Vessel
Tissue
Ifng
Tnf
Ccl5
Csf1
Csf2
Osm
Tgfb1
Clcf1
Il16
Cmtm3
Dock2
Cish
Socs5
Socs3
Socs2
Socs1
Gdf11
Pdgfb
Pdgfa
Vegfc
Vegfb
Vegfa
Tnfsf14
Tnfsf12
Tnfsf10
Tnfsf9
Tnfsf8
Cklf
Ccl25
Ccl9
Ccl4
Ccl3
Xcl1
Normalized Expression
0.00 0.25 0.50 0.75 1.00
C
abs. Numbers
3000
2000
1000
Nephritis Vessel 153 290 1060 427 638 533 1719 3854 204 165 181 28 120
Young Vessel 8 39 223 143 215 291 305 488 175 67 33 7 25
Nephritis Tissue 37 60 347 1166 671 1722 512 679 1393 856 436 283 268
Young Tissue 21 62 255 374 540 357 309 489 1613 750 280 180 131

Figure S1: Extended data on single-cell transcriptomics analysis of ILC populations.

(**A**) Overview of top 5 signature genes for each cell type (cf. Figure 1C), a complete list is provided in Table S1. The color-code corresponds to Figure 1 panels C and E.

(**B**) Normalized expression values of selected cytokines across compartments and disease conditions. If cytokines were also included in the mathematical model, they are printed in bold.

(**C**) Absolute cell numbers of the complete ILC data sets before down-sampling.

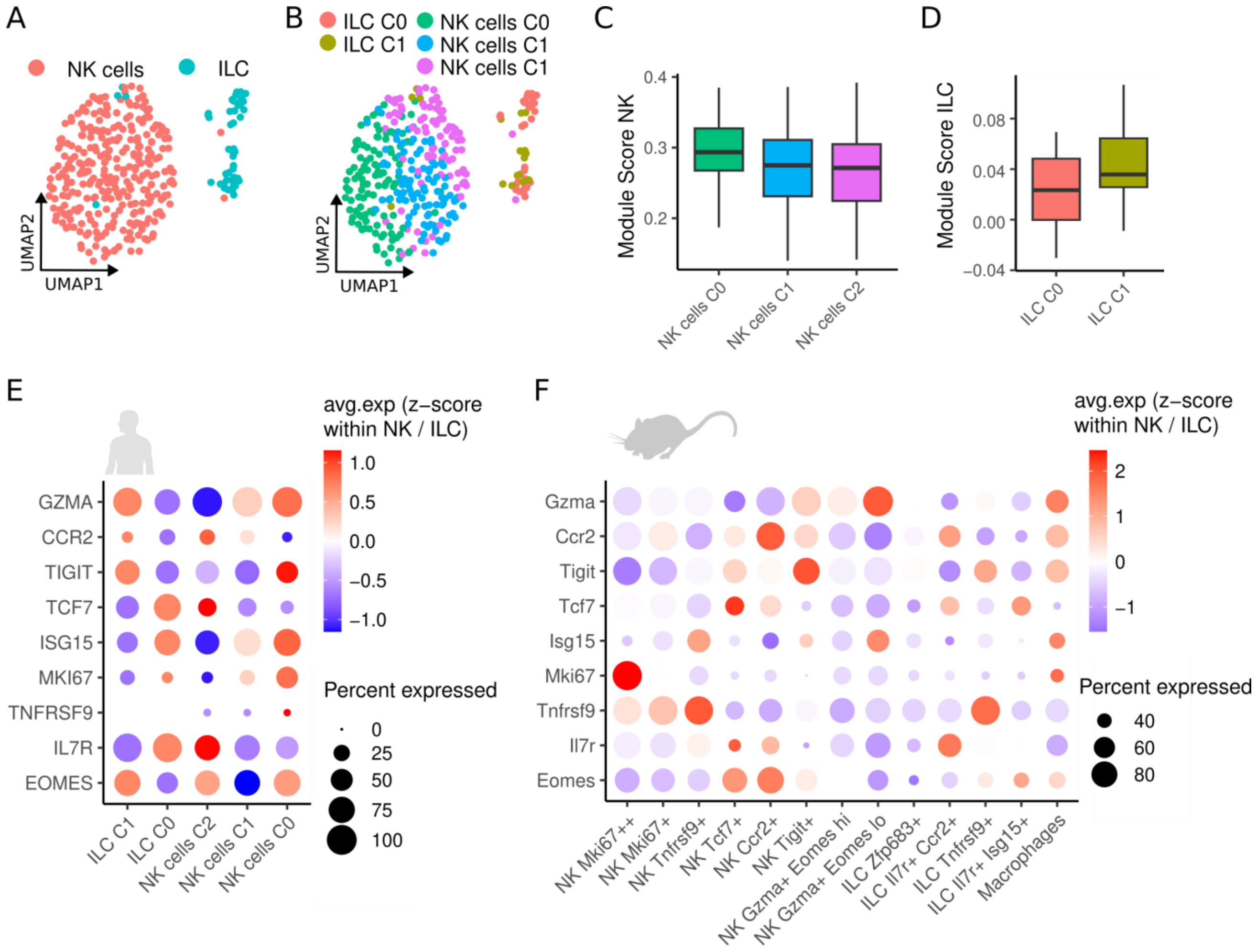

Figure S2: Cross-species validation of NK and ILC activation states in human SLE.

(**A-B**) UMAP visualization of human kidney single-cell RNA-seq data showing coarse annotation and refined clustering into NK cell and ILC subpopulations.

(**C-D**) Module scores for NK cell and ILC activation signatures demonstrate differential activation levels across identified subclusters (middle panels). NK cell activation was scored using the human KEGG "Natural Killer Cell Mediated Cytotoxicity" gene set, while ILC activation was assessed using the human orthologs of the murine activation signature.

(**E-F**) Dot-plots depict scaled average expression (z-score within NK cell or ILC compartment) and percent cells expressing selected activation-associated genes across human subclusters. For comparison, the murine dataset is shown with the same scaling.

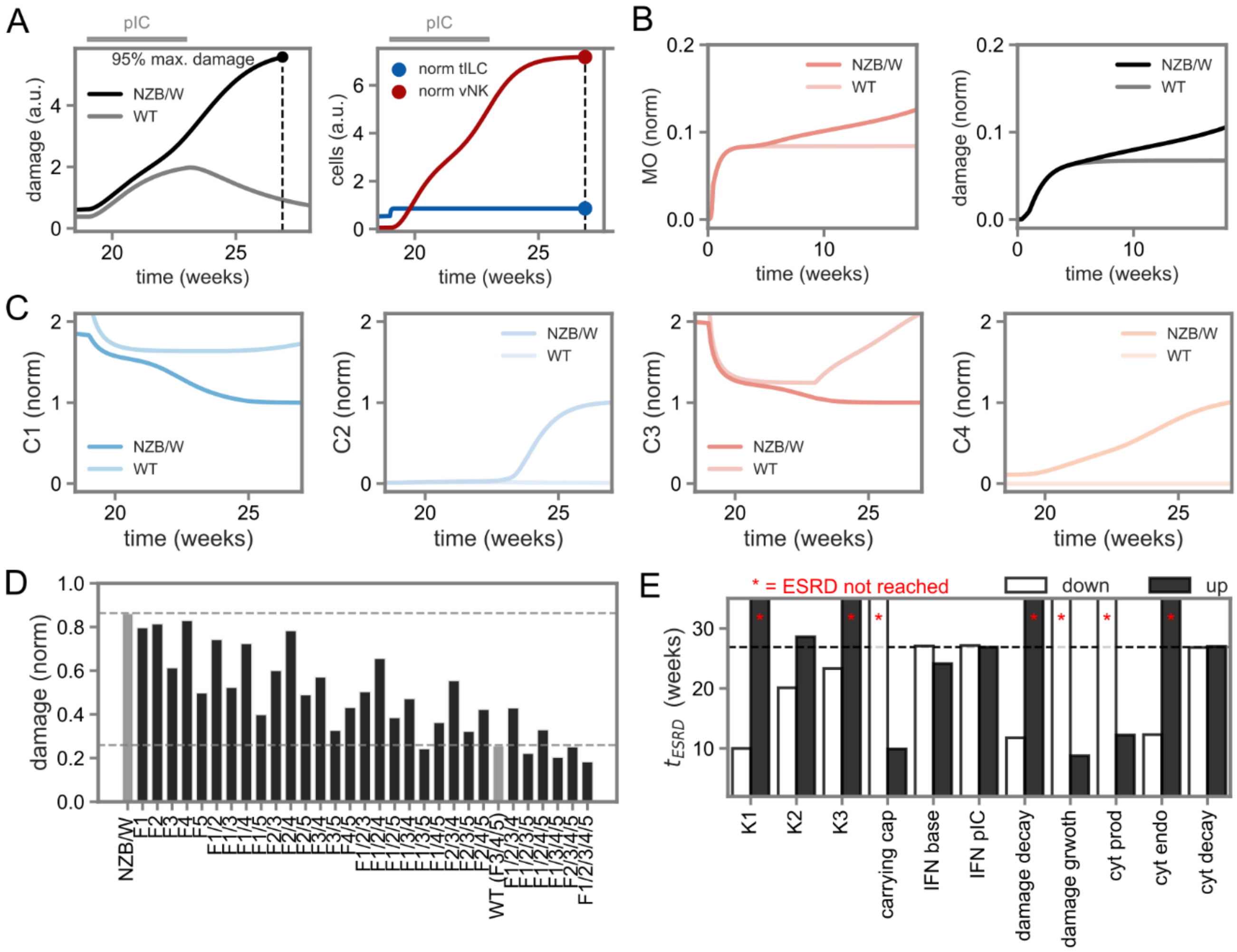

Figure S3: Extended model simulations.

(**A**) End-stage normalization. Model kinetics of all conditions are normalized to the value at time point where 95% of maximum damage in NZB/W condition is reached under poly(I:C) treatment.

(**B**) Monocytes and damage kinetics before poly(I:C) treatment.

(**C**) Cytokine kinetics after normalization.

(**D-E**) Supplementary feedback and sensitivity analysis, analogous to Figure 3D-E.

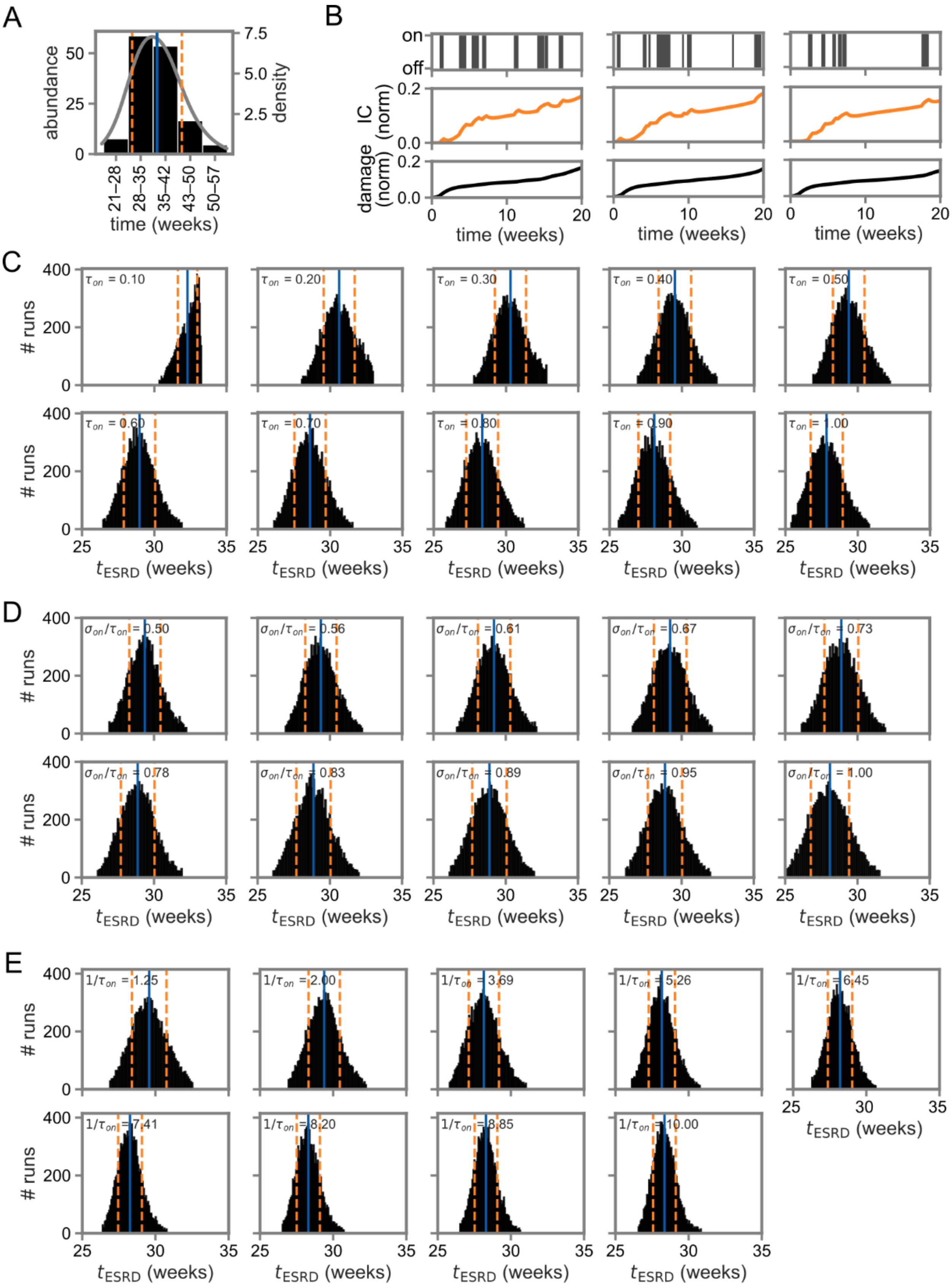
A
abundance
density
time (weeks)
21–28
28–35
35–42
43–50
50–57
B
on
off
IC (norm)
damage (norm)
time (weeks)
C
runs
τ_{on} = 0.10
τ_{on} = 0.20
τ_{on} = 0.30
τ_{on} = 0.40
τ_{on} = 0.50
τ_{on} = 0.60
τ_{on} = 0.70
τ_{on} = 0.80
τ_{on} = 0.90
τ_{on} = 1.00
t_{ESRD} (weeks)
D
σ_{on}/τ_{on} = 0.50
σ_{on}/τ_{on} = 0.56
σ_{on}/τ_{on} = 0.61
σ_{on}/τ_{on} = 0.67
σ_{on}/τ_{on} = 0.73
σ_{on}/τ_{on} = 0.78
σ_{on}/τ_{on} = 0.83
σ_{on}/τ_{on} = 0.89
σ_{on}/τ_{on} = 0.95
σ_{on}/τ_{on} = 1.00
E
$1/\tau_{on}$ = 1.25
$1/\tau_{on}$ = 2.00
$1/\tau_{on}$ = 3.69
$1/\tau_{on}$ = 5.26
$1/\tau_{on}$ = 6.45
$1/\tau_{on}$ = 7.41
$1/\tau_{on}$ = 8.20
$1/\tau_{on}$ = 8.85
$1/\tau_{on}$ = 10.00

Figure S4: Extended analysis of the hybrid model.

(**A**) Estimated distribution of the life-span of NZB/W F1 mice based on literature data (Table 1). The gray curve shows a Gaussian density approximation of the histogram data. The blue line is the mean of the distribution and the orange line the corresponding standard deviation.

(**B**) Three representative instances of the hybrid model, analogous to Figure 4C.

(**C-E**) Histograms showing the time to ESRD and corresponding average (blue) and standard deviation (orange) under varying parameters of the stochastic process, as in Figure 4G.

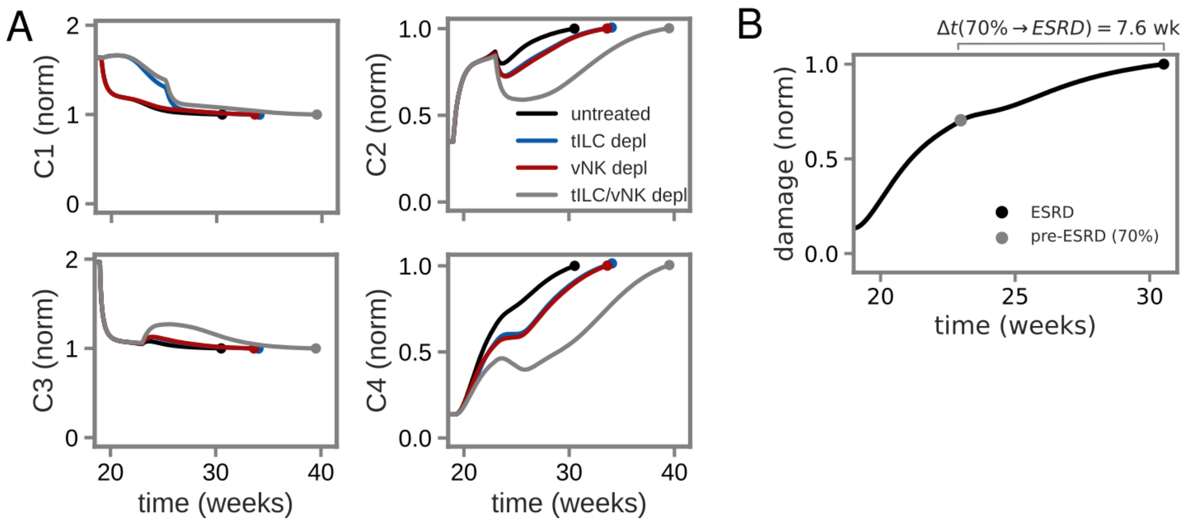

Figure S5: Extended model analysis under depletion conditions.

(**A**) Shown are normalized cytokines kinetics under tILC, vNK and tILC/vNK depletion with depletion strength of 80%, as in Figure 5B.

(**B**) Definition of pre-ESRD as a proxy for proteinuria onset, given by time at which damage attains 70% of ESRD.

Supplementary References

1. Biniaris-Georgallis S-I, Aschman T, Stergioula K, Schreiber F, Jafari V, Taranko A, Karmalkar T, Kasapi A, Lenac Rovis T, Jelencic V, et al. Amplification of autoimmune organ damage by NKp46-activated ILC1. *Nature* (2024)1–3. doi: 10.1038/s41586-024-07907-x
2. Triantafyllopoulou A, Franzke C-W, Seshan SV, Perino G, Kalliolias GD, Ramanujam M, Van Rooijen N, Davidson A, Ivashkiv LB. Proliferative lesions and metalloproteinase activity in murine lupus nephritis mediated by type I interferons and macrophages. *Proc Natl Acad Sci USA* (2010) 107:3012–3017. doi: 10.1073/pnas.0914902107
3. Mortha A, Burrows K. Cytokine Networks between Innate Lymphoid Cells and Myeloid Cells. *Front Immunol* (2018) 9:191. doi: 10.3389/fimmu.2018.00191
4. Barrow AD, Colonna M. Innate lymphoid cell sensing of tissue vitality. *Curr Opin Immunol* (2019) 56:82–93. doi: 10.1016/j.coi.2018.11.004
5. Berry M, Clatworthy MR. Kidney Macrophages: Unique Position Solves a Complex Problem. *Cell* (2016) 166:799–801. doi: 10.1016/j.cell.2016.07.047
6. Liu M, Liang S, Zhang C. NK Cells in Autoimmune Diseases: Protective or Pathogenic? *Front Immunol* (2021) 12:624687. doi: 10.3389/fimmu.2021.624687
7. Lövgren T, Eloranta M-L, Båve U, Alm GV, Rönnblom L. Induction of interferon-alpha production in plasmacytoid dendritic cells by immune complexes containing nucleic acid released by necrotic or late apoptotic cells and lupus IgG. *Arthritis Rheum* (2004) 50:1861–1872. doi: 10.1002/art.20254
8. Liu Z, Davidson A. IFNα Inducible Models of Murine SLE. *Front Immunol* (2013) 4: doi: 10.3389/fimmu.2013.00306
9. Ben Mkaddem S, Benhamou M, Monteiro RC. Understanding Fc Receptor Involvement in Inflammatory Diseases: From Mechanisms to New Therapeutic Tools. *Front Immunol* (2019) 10: doi: 10.3389/fimmu.2019.00811
10. Bournazos S, Gupta A, Ravetch JV. The role of IgG Fc receptors in antibody-dependent enhancement. *Nat Rev Immunol* (2020) 20:633–643. doi: 10.1038/s41577-020-00410-0
11. Majer O, Bourgeois C, Zwolanek F, Lassnig C, Kerjaschki D, Mack M, Müller M, Kuchler K. Type I Interferons Promote Fatal Immunopathology by Regulating Inflammatory Monocytes and Neutrophils during Candida Infections. *PLoS Pathog* (2012) 8:e1002811. doi: 10.1371/journal.ppat.1002811
12. Kwant LE, Vegting Y, Tsang-a-Sjoe MWP, Kwakernaak AJ, Vogt L, Voskuyl AE, van Vollenhoven RF, de Winther MPJ, Bemelman FJ, Anders H-J, et al. Macrophages in Lupus Nephritis: Exploring a potential new therapeutic avenue. *Autoimmun Rev* (2022) 21:103211. doi: 10.1016/j.autrev.2022.103211
13. Atehortúa L, Rojas M, Vásquez GM, Castaño D. Endothelial Alterations in Systemic Lupus Erythematosus and Rheumatoid Arthritis: Potential Effect of Monocyte Interaction. *Mediators Inflamm* (2017) 2017:1–12. doi: 10.1155/2017/9680729
14. Thurley K, Wu LF, Altschuler SJ. Modeling Cell-to-Cell Communication Networks Using Response-Time Distributions. *Cell Syst* (2018) 6:355-367.e5. doi: 10.1016/j.cels.2018.01.016
15. Burt P, Thurley K. Distribution modeling quantifies collective TH cell decision circuits in chronic inflammation. *Sci Adv* (2023) doi: 10.1126/sciadv.adg7668
